# Prevalence and Determinants of Soil-Transmitted Helminthic Infections among School Children at Goro Primary School, South West Shewa, Ethiopia

**DOI:** 10.1155/2020/8612054

**Published:** 2020-08-27

**Authors:** Tigist Tiruneh, Geleta Geshere, Tsige Ketema

**Affiliations:** College of Natural Sciences, Department of Biology, Jimma University, Ethiopia

## Abstract

**Background:**

Soil-transmitted helminths (STH)/geohelminths are human parasitic nematodes which need soil contact for their egg development and become infectious. It is widely prevalent in developing countries. In Ethiopia, too, the same problem exists although the prevalence varies from place to place depending on the presence of risk factors and hygienic status of the community. Therefore, the current study is designed to assess the prevalence and determinants of STH among school children at Goro Primary School of Southwest Ethiopia.

**Methods:**

A cross-sectional study design was employed from April to June 2019. The stool samples were collected in prelabeled, clean, and leak-proof stool cups and examined immediately. Direct wet mount and formalin ether concentration techniques were utilized to detect the STHs in a stool sample collected from all study subjects. A total of 387 stool samples were analyzed. Moreover, community- and individual-level risk factors associated with STH infection were assessed using semistructured questionnaire.

**Results:**

The overall prevalence of soil-transmitted helminth infections observed at the study area was 15.8% (*n* = 61/387). Among these, the most abundant STH parasite was hookworms (*n* = 39/61, 63.93%) followed by *Ascaris lumbricoides* (*n* = 22/61, 36.06%). Factors independently associated with soil-transmitted helminth infections were children from illiterate mother (AOR = 2.3, 95% CI: 1.1-4.8, *P* = 0.021), lack of habit of wearing shoes (AOR = 4.1, 95% CI: 2.0-8.5, *P* < 0.001), lack of frequent handwashing practice before meal (AOR = 2.3, 95% CI: 1.2-4.5, *P* = 0.019), use of unprotected drinking water (AOR = 39, CI:3.9-393, *P* = 0.002), and presence of dirt in their fingernails (AOR = 3.5, 95% CI: 1.8-6.9, *P* < 0.001).

**Conclusions:**

STH infection observed in the study area could be classified into the low-risk area group (according to the World Health Organization classification) calling for none or case-by-case treatment. Thus, enhancing awareness of the community in the study area on how to keep personal hygiene and environmental sanitation is quite important to keep the burden to a controllable level, besides implementation of regular deworming program in the locality.

## 1. Introduction

Soil-transmitted helminth infections remain a significant public health problem of developing countries. According to the World Health Organization (WHO) [1], the STHs of major concern to humans are *Ascaris lumbricoides*, *Trichuris trichiura*, *Necator americanus*, and *Ancylostoma duodenale*. The transmission of STHs is higher in countries where poverty, poor nutrition, inadequate sanitation, overcrowding, failure to put on footwears, poor socioeconomic status, lack of clean drinking water, and minimal health care prevailed [2, 3]. The latest estimates indicate that more than 2 billion people in the world are infected with at least one species of STHs (due to *Ascaris lumbricoides*: 1 billion, *Trichuris trichiura*: 800million and hookworm: 740million) and 4 billion are at risk of acquiring the infections [1, 4]. The highest prevalence occurs in areas where sanitation is inadequate and water supplies are unsafe [1, 4]. WHO [1] reported that the number of children who received preventive chemotherapy every year was only 200 million out of 600 million school-age children (SAC). According to the Federal Ministry of Health of Ethiopia (FMoH) [5], both schistosomiasis and STHs are endemic in Ethiopia and represent significant health burdens. The number of people living in STH endemic areas is estimated to be 81 million, which comprised 9.1 million preschool-aged children, 25.3 million school-aged children, and 44.6 million adults [5]. The number of individuals living in areas qualifying for STH mass drug administration is 56.7 million, comprising of 4.6 million preschool children, 17.7 million school-age children, and 31.32 million adults [5]. Large-scale and successful control activities implemented during 2001–2010 demonstrate the feasibility of large-scale deworming, and these experiences have informed the development of tools to facilitate the work of control managers. The major limitations of the national program to fight against the STHs were lack of compiled information about the prevalence of STHs in all districts/localities, and lack of consistency and just relying only on mass drug administration (MDA) [1]. Therefore, the aim of this study was to assess the prevalence of STH infections and its associated risk factors among school-age children at one of the potential endemic districts in Ethiopia.

## 2. Methods

### 2.1. Description of the Study Area

**Figure 1:**
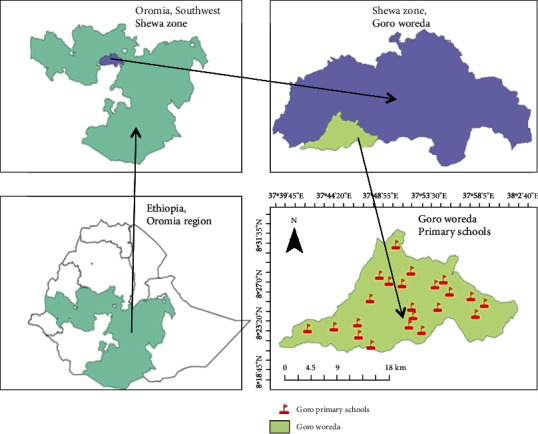
Map of the study area.

The study was conducted at Goro Elementary School located in Goro Wereda, Oromia Regional State, Ethiopia. It is located 135 kms from Addis Ababa, the capital city of Ethiopia, and 21 kms from Woliso town to Southwest (Figure 1). The livelihood of the inhabitants mainly depends on subsistence farming, with teff and maize being the commonly produced cereals. The altitude and temperature of the area fall within the ranges of 1660-1906 meters above sea level (masl) and 9-30°C, respectively. The average annual rainfall is 800-1600 mm.

### 2.2. Study Design and Sample

A cross-sectional study design was conducted to determine the prevalence and risk factors of soil-transmitted helminths at Goro Primary School, Goro Wereda, Southwest Shewa from April to June 2019. A total of 1242 children (628 boys and 614 girls) were enrolled at Goro Primary School. The sample size of this study was determined using a single population proportion formula [6]. Considering the critical value of the normal distribution at *α*/2 at 95% = 1.96, prevalence of 50%, and the margin of error of ±0.05, a total of 384 samples were used. Also, by adding 5% nonresponse rate, the final sample size was adjusted to 403 students. To select each study subject, the students were first stratified according to their educational level (Grade 1 to Grade 8). Then, the study subjects were selected using systematic random sampling (every 3^rd^ number on the class attendance sheets as a sampling frame). From the 403 students, only two of them refused to participate, and 14 students did not volunteer to give sample. Therefore, fecal samples were obtained only from 387 students. All selected school children, who consented to participate in the study and attended the school during the study period, were enrolled in the study. Whereas, those students who have taken any antihelminthic drug before two weeks of the study period were excluded from the study.

### 2.3. Data Collection Procedure

Sociodemographic characteristics and associated risk factors such as age, sex, residence, religion, child educational level availability of latrine house, mother's educational level, shoe wearing habit, handwashing practice before meal, washing habit after toilet, source of drinking water, presence of dirt in the fingernails, and playing with soil in the vicinity were collected by trained data collectors using pretested questionnaires. The questionnaire was first prepared in English and then translated into the local language, Afan Oromo, which was administered by a researcher. A stool sample was collected from each study subject using labeled, clean, and leak-proof stool cup.

### 2.4. Fecal Sample Analysis

A drop of saline and a small amount of faces were placed on a microscopic slide using applicator stick, mixed and covered with coverslip. Then, the samples were examined under the light microscope at a magnification power of 400x. The remaining portion of the tool samples (~1 g) were preserved in 10% formalin for sedimentation concentration technique [7] when the result of direct wet mount turned negative. Accordingly, the preserved sample was strained through gauze, mixed with saline, and centrifuged at 2000-2500 rpm for a minute. The supernatant was decanted and washed again with tape water. About 10 mL of 10% formalin was then added to the sediment and mixed thoroughly. This was followed by the addition of 3 mL of ether, shacked vigorously for 30 seconds, and centrifuged at 1500 rpm for a minute. The three top layers were decanted carefully, and the sediment was removed with a cotton swab and examined microscopically under high-power magnification. Laboratory procedures were performed in Goro Health Center by two laboratory technologists.

### 2.5. Data Analysis

The questionnaire was checked for completeness, coded, and entered into excel and cleaned. It was exported to and analyzed using SPSS (statistical package for social sciences) version 20.0 statistical software. Descriptive statistics was utilized to summarize the sociodemographic profile of the study participants. Bivariate and multivariate logistic regression models were used to determine the association between dependent and independent variables; 95% confidence interval (CI) was used for all analyses, and significant level was considered at *P* < 0.05.

## 3. Results

### 3.1. Sociodemographic Characteristics of the Study Participants

Out of 403 randomly selected students, 387 (96.3%) were volunteers to participate in the study and provided stool sample. The largest number of students, 268 (70%), were from the age group of 11–16 years, and the remaining 119 (30%) were aged between 6 and 10 years. The study participants consisted of 188 (48.6%) males and 199 (51.4%) females. About 185 (47.8%) were urban residents, while 202 (52.2%) live in rural area. Regarding the grade level, 215 (55.6%) were in grades 1-4, and 172 (44.4%) were in grades 5-8. The majority of the students, (71.1%, *n* = 275), belong to households with family size greater than five, and 112 (28.9%) belong to family size less than five (Table 1).

### 3.2. Prevalence and Associated Risk Factors of Soil-Transmitted Helminths

**Figure 2:**
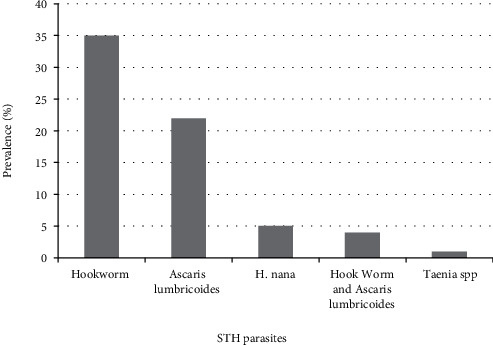
Prevalence of soil-transmitted helminthic parasites among school-age children at Goro Elementary School in Goro Wereda, South West Shewa, 2018/19.

From the 387 stool specimens examined, the prevalence of soil-transmitted helminthic parasite was 15.8% (*n* = 61). Out of these, 57 were infected with either Hookworms or *A. lumbricoides*, and only four students had double helminthic infection, infection with Hookworms and *A. lumbricoides* (Figure 2). Other intestinal protozoan parasites, including *Giardia intestinalis* and *Entamoeba histolytica/E. dispar* were found infecting 11 and 6 children, respectively.

### 3.3. Determinants of STH Infections

Individual-level characteristics including sociodemographic characteristics and associated risk factors such as age, sex, residence, religion, grade level, and availability of latrine house were not significantly associated with infection of STHs (*P* > 0.05). Other variables such as mother's educational level, shoe wearing habit, handwashing practice before meal, washing habit after toilet, source of drinking water, presence of dirt in the fingernails, and playing with soil in the vicinity were significantly associated (*P* < 0.05) with the infection of STHs and found as individual-level risk factors (Table 2).

Individual-level risk factors were further evaluated by multivariable analysis for their predictive potential. Accordingly, among these characteristics, family size, grade of children, availability of latrine house, habit of handwashing after toilet, and playing with soil did not show significant association (*P* > 0.05) with STH infection (Table 3). The prevalence of STH infections was significantly associated (*P* < 0.05) with shoe wearing habits in which children infrequently wearing shoes were four times more likely to acquire the STH infection as compared to children who wear shoes always (AOR: 4.1, 95% CI: 2.0-8.5, *P* < 0.001). The irregular handwashing habit before meal had also significant association with STH infection as compared to individuals regularly washing their hands before meal (AOR = 2.3; 95% CI: 1.2-4.5; *P* = 0.019). Those children infrequently washing their hands after toilet had also significantly higher risk of having STH infection (COR = 5.6; 95% CI: 1.9-16.5; *P* < 0.05) but did not show significant association in multivariable analysis (AOR = 1.5; 95% CI: 0.4-5.2; *P* > 0.05). Family size had also significant association with STH infection (COR = 2.3; 95% CI: 1.1-4.8; *P* < 0.05) but did not show significant association in multivariable analysis (AOR = 2.1; 95% CI: 0.90-4.6; *P* > 0.05). The STH infection was three times higher (AOR: 3.5, 95% CI: 1.8–6.9) in children who had dirt in their fingernails as compared to those who did not. Drinking water from unprotected source had a strong association with STH infection in which individuals who use unprotected water were more likely to be STH positive (AOR = 39.0; 95% CI: 3.9-393, *P* < 0.05). STH infection was significantly associated with frequent habit of playing with soil (COR = 2.6, 95% CI: 1.5-4.7, *P* < 0.05) but did not show significant association in multivariable analysis (AOR = 1.5, 95% CI: 0.7-3.2, *P* > 0.05) (Table 3).

## 4. Discussion

Due to differences with the risk factors at different localities, environmental sanitation, and culture of the community among regions, the prevalence and distribution of Intestinal Parasitic Infection (IPI) varies in Ethiopia [8]. The study findings showed that STH infections are still major public health concern among children in Goro district with 15.8% (61/387) of participants infected with at least one STH species. Out of the three STH species, hookworm infection was the most predominant (9.1%), followed by infection by *A. lumbricoides* (5.7%). The prevalence of STH infection observed in the current study was relatively lower than reports from other districts of the same country, including Jimma town (45.6%), Durbete Town (54.9%), and Tilili town (44.2%) [9–11]. According to WHO's [1] classification of endemic areas of STH, there are three categories in line with the application of mass drug administration (MDA). These are high transmission (where prevalence is >50%), moderate transmission (where prevalence is between 20% and 50%), and low transmission (where prevalence is <20%). Thus, the current study area could be classified under the low-risk area group which calls for none, but a case-by-case treatment. In fact, in the study area, there is mass chemotherapy usually undertaken twice a year. Unlike other developing regions, the low prevalence of STH observed in the study area might partly be due to the contribution of the deworming or mass drug administration program that targeted only children aged between 2 and 5 years. According to information obtained from the district health officials, in 2018/19 only, a total of 5405 children already received antihelminthic drugs in the deworming campaign. As children actively interact with each other, their playful behavior exposes them to contaminated soil, water, and food; hence, the likelihood of rereinfection is high in older children. Currently, major deworming program has been implemented in different parts of the country, and this has shown a significant reduction of helminthic infections in most parts of the country such as Gurage zone (9.5%), Ambo Town (12.6%), and Babile Town (0.47%) [11–13].

This study indicated that Hookworms (9.1%), *A. lumbricoides* (5.5%), *H. nana* (1.3%), and Taenia spp. (0.25) were the intestinal helminths parasitizing school children of Goro Primary School. The prevalence of hookworm observed in the current study was significantly higher than other intestinal helminths which might be due to the fact that most infected children were from rural area and they might wear shoes only sometimes and played or walked over loamy soils and cultivated fields without shoes. As a result, children walking barefooted on soil contaminated with fecal matter could be exposed to the infective larval stages of the hookworm parasite [9]. The most common combination of STHs in this study is hookworms and *A. lumbricoides* which agrees with report by Alelign and colleague [9] from other part of the same country. This is because *Ascaris* and hookworms are the most abundant and the main species that infect people [11–15]. Effects of high load of these infections usually result in malnutrition, iron deficiency anemia, malabsorption syndrome, intestinal obstruction, respiratory complications, poor weight gain, and impaired cognition in children [2, 16].

The study identified key predictors of STH in children, which included sociodemographic factors, use of unprotected water, not washing hands before feeding, walk without shoe, and dirt in the fingernail. This is probably due to poor awareness on part of both children and their parents about the fecal-soil-oral transmission of helminths through their unwashed hands, or due to lack of handwashing facilities [16]. Handwashing is one of the important parameters which intervene the fecal-oral transmission infections. Thus, handwashing must be practiced well before meal and after use of toilet. The overall prevalence of intestinal parasitosis is greater among children aged between 5 and 14 years. This is because at this age changes in children behavior further expose them to conditions that favor the transmission of soil-transmitted helminthic infections [17].

Other factors found associated with infection in this study were walking barefooted. This might be due to children's engagement in agricultural activities to help their parents, which in turn, exposed them to infective stages of the parasites [8]. Hookworm infections were higher in children not wearing shoes. This indicated that wearing shoes have great importance in protecting hookworm infection [2]. Using unprotected water had a significant association with Ascaris infection. Accordingly, using protected water and avoiding contamination and keeping self-hygiene could contribute to a reduction of Ascaris infection. This is because unprotected water is not usually covered, and infective stage of Ascaris could be introduced into the water through flood or direct damping of sewage into the water, hence, are susceptible to contamination with human and animal feces containing infective stages of the parasite.

## 5. Limitation of the Study

This study has certain limitations that need to be considered while interpreting the results. Most of the questions were self-reported which could possibly compromise the accuracy of the information collected from the study participants. In addition, supplementing the parasitological diagnostic approaches with other more sensitive techniques such as the Kato Katz method could have enhanced the probability of detecting more STH.

## 6. Conclusion

This study ascertains that STH infections were detected among school children at Goro Primary School. The most common species responsible for the observed infections were hookworm followed by *A. lumbricoides*. The school children are at low risk of infection with STHs according to WHO classification of prevalence, as the observed prevalence was <20% that needs only case-by-case treatment. Among the risk factors associated with STH infections were shoes wearing habit, lack of handwashing before meal, unprotected water sources, and dirt in fingernails. We suggest health education on basic sanitation to the community in the study area.

## Figures and Tables

**Table 1:** Sociodemographic characteristics of the study participants, among school-age children at Goro Elementary School, Southwest Ethiopia, 2019.

Variable	Alternative	Frequency (%)
Age	6-10	119 (30.0)
	11-16	268 (70.0)

Sex	Female	199 (51.4)
	Male	188 (48.6)

Family size	≤5	112 (28.9)
	>5	275 (71.1)

Residence	Urban	185 (47.8)
	Rural	202 (52.2)

Religion	Orthodox	205 (53.0)
	Muslim	128 (33.1)
	Protestant	49 (12.6)
	Wakefeta	5 (1.3)

Grade	1-4	215 (55.6)
	5-8	172 (44.4)

**Table 2:** Prevalence of STHs and individual-level associated risk factors among school-age children at Goro Elementary School, Southwest Ethiopia, 2018/19.

Variable	Alternative	STH status		COR (95% CI)	*P* value
		Participants (%)	Positive (%)		
Age	6-10	119 (30.7)	22 (18.5)	1.4 (0.8-2.5)	0.259
	11-15	268 (69.3)	39 (14.5)		

Sex	Female	199 (51.4)	34 (17.1)	1.2 (0.7-2.1)	0.463
	Male	188 (48.6)	27 (14.4)		

Mother's educational status	Illiterate	133 (34.4)	18 (13.5)	2.3 (1.1-4.8)	0.021∗
	Literate	254 (65.6)	43 (16.9)		

Family size	≤5	112 (28.9)	10 (8.9)	1.3 (0.7-2.4)	0.385
	>5	275 (71.1)	51 (18.6)		

Residence	Rural	202 (52.2)	35 (17.3)	1.3 (0.7-2.2)	0.378
	Urban	185 (47.8)	26 (14.1)		

Religion	Orthodox	205 (53.0)	31 (15.1)		
	Muslim	128 (33.1)	20 (15.6)	1.0 (0.6-1.9)	0.901
	Protestant	54 (13.95)	10 (2.58)	1.3 (0.6-2.9)	0.576

Grade	1-4	215 (55.6)	40 (18.6)	1.6 (0.9-2.9)	0.088
	5-8	172 (44.4)	21 (12.2)		

Latrine availability and using practice	No	39 (10.1)	10 (25.6)	2.0 (0.9-4.4)	0.079
	Yes	348 (89.9)	51 (14.7)		

Shoe wearing habit	Sometimes	154 (39.8)	46 (29.9)	6.2 (3.3-11.6)	*P* < 0.001^∗^
	Always	233 (60.2)	15 (6.4)		

Handwashing habit before meal	Sometimes	96 (24.8)	28 (29.2)	3.2 (1.8-5.7)	*P* < 0.001^∗^
	Always	291 (75.2)	33 (11.3)		

Handwashing habit after toilet	Not at all	152 (39.3)	31 (20.4)	5.6 (1.9-16.5)	0.002^∗^
	Only with water	143 (36.9)	26 (18.2)	4.9 (1.6-14.5)	0.004^∗^
	Wash with water and soap	92 (26.8)	4 (4.4)		

Source of drinking water	Protected	382 (98.7)	57 (14.9)		
	Unprotected	5 (1.3)	4 (80.0)	22.8 (2.5-207.8)	0.006^∗^

Presence of dirt in fingernail	No	221 (57.1)	20 (9.1)		
	Yes	166 (42.9)	41 (24.7)	3.3 (1.8-5.9)	*P* < 0.001

Habit of playing with soil	No	210 (54.3)	21 (10.0)		
	Yes	177 (45.7)	40 (22.6)	2.6 (1.5-4.7)	0.001^∗^

NB: COR: crude odds ratio; CI: confidence interval; ^∗^significant at *P* < 0.05.

**Table 3:** Factors associated with STH among school-age children at Gora Elementary School, Southwest Ethiopia, 2019.

Variable	Alternative	Participants (%)	Positive (%)	AOR (95% CI)	*P* value
Family size	>5	275 (71.1)	51 (18.6)	2.1 (0.9-4.6)	0.072
	≤5	112 (28.9)	10 (8.9)		

Grade	1-4	215 (55.6)	40 (18.6)	1.0 (0.4-2.0)	0.841
	5-8	172 (44.4)	21 (12.2)		

Latrine available	No	39 (10.1)	10 (25.6)	1.4 (0.6-3.6)	0.426
	Yes	348 (89.9)	51 (14.7)		

Shoe wearing habit	Sometimes	154 (39.8)	46 (29.9)	4.1 (2.0-8.5)	0.000^∗^
	Always	233 (60.2)	15 (6.4)		

Hand wash before meal	Sometimes	96 (24.8)	28 (29.2)	2.3 (1.2-4.5)	0.019^∗^
	Always	291 (75.2)	33 (11.3)		

Hand wash after toilet	Not at all	152 (39.3)	31 (20.4)	1.5 (0.4-5.2)	0.560
	Only with water	143 (36.9)	26 (18.2)	2.7 (0.8-9.0)	0.097
	With water and soap	92 (26.8)	4 (4.4)		

Drinking water source	Unprotected	5 (1.3)	4 (80.0)	39.0 (3.9-393)	0.002^∗^
	Protected	382 (98.7)	57 (14.9)		

Dirt in fingernail	Yes	166 (42.9)	41 (24.7)	3.5 (1.8-6.9)	0.000^∗^
	No	221 (57.1)	20 (9.1)		

Play with soil	Yes	177 (45.7)	40 (22.6)	1.5 (0.7-3.2)	0.337
	No	210 (54.3)	21 (10.0)		

NB: AOR: adjusted odds ratio; CI: confidence interval; ^∗^significant at *P* < 0.05.

## Data Availability

The data used to support the findings of this study are all included/available in the manuscript.
